# Editorial: Image and geometry analysis for brain informatics

**DOI:** 10.3389/fninf.2023.1174531

**Published:** 2023-04-28

**Authors:** Yimin Wang, Zhi Zhou, Min Liu

**Affiliations:** ^1^Guangdong Institute of Intelligence Science and Technology, Hengqin, China; ^2^Microsoft, Redmond, WA, United States; ^3^Hunan University, Changsha, China; ^4^Research Institute of Hunan University, Chongqing, China

**Keywords:** brain imaging data, bioimage processing, geometry analysis, neuronal morphology, image registration

Advancements in brain science research have led to the high-throughput generation of vast amounts of data, with the multi-modality, multi-resolution imaging and geometry data being a vital part (Peng et al., 2015, 2021; Ecker et al., 2017; Regev et al., 2017; Winnubst et al., 2019; Callaway et al., 2021). To gain a deeper understanding of the complexities of the brain, it is crucial to properly process and analyze such data. However, due to the overwhelming amount, large scale, and complex nature of the data, these tasks pose significant challenges that need to be overcome through novel and advanced methods. The research articles included in this collection provide up-to-date findings and insights on several issues related to the topic of *image and geometry analysis for brain informatics*, such as image registration, geometrical model generation, and super-resolution reconstruction.

Sun et al. proposed a deep self-calibration-based progressive image registration strategy to address large deformations while avoiding information loss and additional parameters. The method leverages cascaded networks and a novel hierarchical registration strategy to achieve more accurate multi-scale progressive registration and dynamic dataset augmentation. The proposed method was evaluated on optical and MRI image datasets using specific performance criteria and was compared to several state-of-the-art approaches for deformable image registration. The results demonstrated improvements in performance over the existing methods, indicating the effectiveness of the proposed approach.

Han et al. provided a comprehensive survey on the use of Generative Adversarial Networks (GANs) for mono- and cross-modal biomedical image registration. They identified four categories of GAN-based methods based on implementation strategies: modality translation, symmetric learning, adversarial strategies, and joint training. For each category, the authors summarized and discussed the specific techniques and approaches used, as well as the main contributions, advantages and disadvantages. In addition, the authors outlined four interesting research directions for future studies and analyzed the statistics of the references from different perspectives to reveal trends in GAN-based biomedical image registration studies.

Zhu et al. developed a technique for surface meshing of a neuron's plasma membrane. The methodology utilizes graph information of the cell and vertex-based diameter information to construct 2D manifolds represented by a triangular surface grid. The key idea is to begin with the soma sphere and deform the mesh along the graph provided by the input data. The technique involves convolving a spherical field along the center-lines of the morphology skeleton to create the facets of the membrane. To minimize branching artifacts and build a smooth surface, a local mapping technique is adopted to update the membrane vertices within a small region of interest. Mesh tessellation is adjusted by a set of quasi-uniform rules, taking into account the surface curvature of the mesh and the morphological characteristics of the neuron. The authors compared their method to existing techniques and demonstrated that their approach is more reliable and produces higher quality meshes.

Shin et al. employed a convolutional neural-network-based architecture for enhancing the quality of Diffusion-Weighted Imaging (DWI) via super-resolution. This method resulted in an image that was much closer to the target image than the interpolation method. Moreover, the similarity indices were significantly enhanced, as indicated by the improved peak signal-to-noise ratio (PSNR) and structural similarity index measure (SSIM). Additionally, the diffusion index mapping reconstructed by Generalized Q-Sampling Imaging (GQI) exhibited improved performance, with clearer visualization of the ventricles and white matter regions. The proposed super-resolution method has potential applications in post-processing low-resolution images, and can offer more accurate fiber geometry description on a sub-voxel scale.

In summary, further exploration of the brain relies on the continuing development of brain informatics techniques. There exists a broad scope of research problems in the field of brain science regarding the computation and analysis of imaging and geometry data that require to be further addressed. Although this collection of research articles provides valuable insights into several detailed problems, many issues are yet to be covered. Further investigation of the problems in this field will lead to the development of more powerful computational tools for us to understand the brain.

## Author contributions

YW, ZZ, and ML wrote the manuscript. All authors read and approved the submitted version.
